# 1807. Tracking antibiotic de-escalation using a novel metric, days of antibiotic spectrum coverage (DASC)

**DOI:** 10.1093/ofid/ofac492.1437

**Published:** 2022-12-15

**Authors:** Hiroyuki Suzuki, Brett Heintz, Daniel J Livorsi, Eli N Perencevich, Michihiko Goto

**Affiliations:** University of Iowa Carver College of Medicine, Iowa City, Iowa; Iowa City VA Medical Center, Iowa City, Iowa; University of Iowa Carver College of Medicine, Iowa City, Iowa; University of Iowa/Iowa City VAMC, Iowa City, Iowa; University of Iowa/Iowa City VAMC, Iowa City, Iowa

## Abstract

**Background:**

In the United States, days of therapy (DOT) is the currently preferred method for measuring antibiotic consumption. A major disadvantage of DOT is that it does not account for the antimicrobial’s spectrum. Antibiotic de-escalation is therefore not well-captured by DOT. To overcome the limitation of DOT, we recently proposed a new metric, days of antibiotic spectrum coverage (DASC). DASC is calculated by multiplying DOT and the antibiotic spectrum coverage (ASC) score, which was assigned based on the activities of each antibiotic agent for 11 wild type bacteria and 5 acquired resistance patterns. Here we evaluate the performance of DASC using antibiotic consumption data where the main antimicrobial stewardship program (ASP) activity is daily prospective audit-and-feedback of all inpatients on antibiotics.

**Methods:**

We analyzed the retrospective cohort of inpatient antibiotic use at Iowa City VA Health Care System from 2017 to 2021. Total monthly DOT was the aggregation of monthly DOT for each antibiotic. DASC for each antibiotic was calculated by multiplying ASC for each antibiotic to DOT. DASC for each antibiotic was aggregated to total monthly DASC. Days-present was used as a denominator for both metrics. Trends of total monthly DOT and DASC were analyzed by simple linear regression.

**Results:**

There was no significant trend in monthly DOT (Figure A: beta= 0.30, 95%CI: -0.67 to 1.27, p=0.55), but there was a significant downward trend in monthly DASC (Figure B: beta= -6.89, 95%CI: -12.7 to -0.98, p=0.03).

**Figure:**
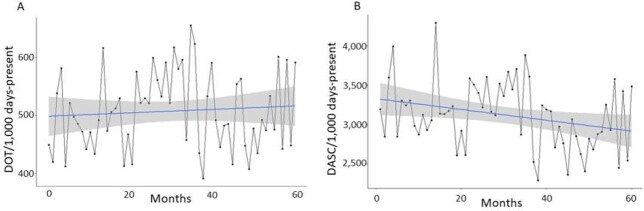

**Conclusion:**

While a significant downward trend was seen using monthly DASC, no trend was observed using monthly DOT. In contrast to DOT, which captures antibiotic consumption without considering the spectrum, DASC captures both antibiotic consumption and spectrum, suggesting DASC can add another dimension in tracking inpatient antibiotic use. DASC is easily calculated if DOT for each antibiotic is available. Metrics that don’t capture antimicrobial spectrum may not be able detect improvements in avoiding unnecessary use of broad-spectrum antimicrobials, an important target of ASPs.

**Disclosures:**

**Daniel J. Livorsi, MD**, Merck & Co.: Grant/Research Support **Michihiko Goto, MD MSCI**, Merck & Co.: Grant/Research Support.

